# Identification of core noncoding RNA-associated competing endogenous RNA networks in renal fibrosis via whole-transcriptome sequencing

**DOI:** 10.1080/0886022X.2025.2522973

**Published:** 2025-06-25

**Authors:** Yizhen Chen, Meng Cheng, Rong Dai, Weili Wang, Yonghao Sang, Liuting Wei, Yilin Gao, Wei Zhang, Yiping Wang, Lei Zhang

**Affiliations:** ^a^ First Clinical Medical College, Anhui University of Chinese Medicine, Hefei, China; ^b^ Department of Nephrology, The First Affiliated Hospital of Anhui University of Chinese Medicine, Hefei, China

**Keywords:** Renal fibrosis, noncoding RNA, whole-transcriptome sequencing, ceRNA networks

## Abstract

**Objective:**

This study aimed to investigate noncoding RNA (ncRNA) expression changes in renal fibrosis (RF) models induced by three distinct etiologies using whole-transcriptome RNA sequencing, identify overlapping differentially expressed (DE) ncRNAs, construct core competing endogenous RNA (ceRNA) networks, and explore their role in RF.

**Methods:**

Three RF rat models, 5/6 nephrectomy, adenine, and unilateral ureteral obstruction, were established. DE RNAs were identified through sequencing and validated by real-time quantitative polymerase chain reaction. ceRNA and RNA-binding protein (RBP) networks were visualized using Cytoscape. Core ceRNA axes were validated with dual-luciferase assay, RNA fluorescence *in situ* hybridization, western blot, immunofluorescence, and immunohistochemistry. Enrichment analysis was performed to explore potential functions.

**Results:**

Sequencing analysis revealed significant dysregulation of ncRNAs in all models compared to the normal group. Intersection analysis identified 215 mRNAs, 19 lncRNAs, and 247 circRNAs as overlapping DE RNAs. lncRNA-based ceRNA networks comprising 7 lncRNAs, 8 miRNAs, and 21 mRNAs, and circRNA-based networks comprising 13 circRNAs, 29 miRNAs, and 41 mRNAs were constructed. The TCONS_00008870/circRNA_3140–miR-466b-3p–Adamts2 axis was identified as a key regulatory pathway. Enrichment analysis showed significant pathways including Rap1 signaling, extracellular matrix–receptor interaction, and PI3K-Akt signaling, with RBPs enriched in RNA binding and ferroptosis.

**Conclusion:**

By integrating data from three distinct models, we identified conserved ceRNA axis—TCONS_00008870/circRNA_3140–miR-466b-3p–Adamts2—potentially modulating fibrotic progression in renal tissue.

## GRAPHICAL ABSTRACT

**Figure:**
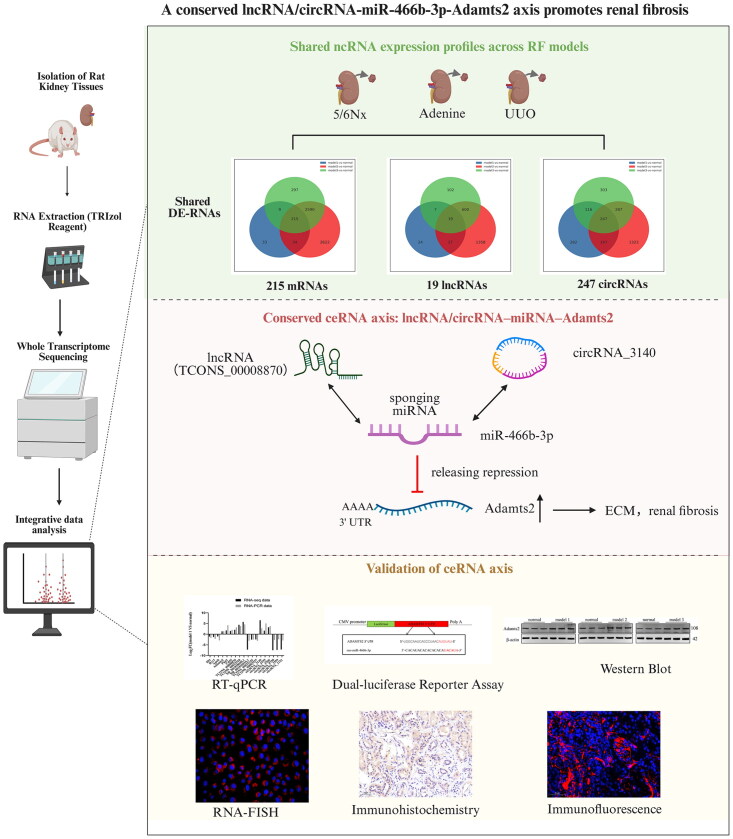

## 1. Introduction

Renal fibrosis (RF), which is characterized by the excessive deposition of the extracellular matrix (ECM), leads to the formation of scar tissue [1,2]. A common pathological feature of various progressive kidney diseases, RF can progress to renal failure, placing a substantial health burden on patients [3,4]. Loss of nephron units, drug-induced toxicity, and obstructive mechanical damage result in genetic alterations in the kidney, promoting the development of RF. Three classic animal models of RF—5/6 nephrectomy (5/6 Nx), adenine-induced kidney injury, and unilateral ureteral obstruction (UUO)—simulate chronic kidney disease (CKD), gouty nephropathy, and obstructive nephropathy, respectively, in clinical settings [5–7]. These models, which exhibit good reproducibility, have been extensively used in basic research on RF; however, the molecular mechanisms of RF at the genomic level remain to be explored further.

Noncoding RNAs (ncRNAs) refer to RNA molecules that do not encode proteins, primarily microRNAs (miRNAs), long noncoding RNAs (lncRNAs), and circular RNAs (circRNAs) [8]. For the discovery of miRNAs and their role in post-transcriptional gene regulation, Victor Ambros and Gary Ruvkun were awarded the 2024 Nobel Prize in Physiology or Medicine [9]. miRNAs directly bind to complementary sequences in the 3′ untranslated region (UTR) of target mRNAs, inhibiting their expression [10]. lncRNAs, which are longer than 200 nucleotides, possess diverse structures and functions [11], while circRNAs regulate gene expression, transcription, and translation by sequestering miRNAs [12]. lncRNAs and circRNAs form competing endogenous RNA (ceRNA) networks by competing with mRNAs for the same microRNA response elements (MREs), thus regulating the expression of transcripts [13]. Increasing evidence indicates that ncRNAs can act as ceRNAs in RF. For example, lncRNA small nucleolar RNA host gene 14 (SNHG14) alleviates kidney injury and fibrosis by regulating the miR-30e-5p/SOX4 axis [14]. Circ-0000953 mitigates podocyte damage by targeting miR-665-3p and regulating autophagy-related genes [15]. Moreover, ncRNA expression profiles and ceRNA networks have been identified in UUO, unilateral ischemia-reperfusion injury animal models, and patients with diabetic nephropathy [16–18]. Additionally, RNA-binding proteins (RBPs) can influence the stability, transcription, and translation efficiency of ncRNAs by binding to them, thereby affecting the function of the ceRNA networks [19]. Research has shown that RBPs play crucial regulatory roles in cardiac regeneration [20], immune regulation [21], and inflammatory diseases [22]. However, most existing studies focus on single models and have yet to uncover the common molecular mechanisms and potential regulatory pathways across different RF models.

Therefore, this study aimed to comprehensively analyze ncRNA expression profiles in three distinct RF models using whole-transcriptome sequencing. Uniquely, by integrating differential expression and network analyses across these models, we sought to identify conserved ceRNA regulatory mechanisms that may underlie shared fibrotic processes. This cross-model approach addresses a critical gap in the current literature, where most studies focus on single models and fail to uncover universally relevant regulatory axes.

## 2. Materials and methods

### 2.1. Animal experiment

**Figure 1.**
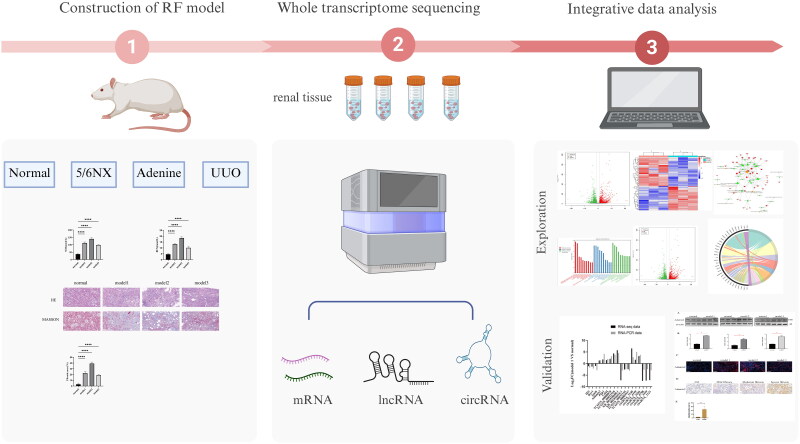
Flowchart of the entire experimental protocol.

A detailed flowchart of the study design is shown in Figure 1. Forty specific pathogen-free -grade male Sprague Dawley rats (200 ± 20 g, 8 weeks old) were purchased from Pizhou Dongfang Breeding Co., Ltd. (production license number: SCXK(Su)2022-0005). The animals were acclimatized for 1 week before the experiment under appropriate room temperature and humidity conditions. The 40 rats were randomly divided into four groups: the normal group (*n* = 10), the model 1 group (5/6 Nx, *n* = 10), the model 2 group (adenine-induced, *n* = 10), and the model 3 group (UUO, *n* = 10). In model 1, the upper and lower poles of the left kidney were removed, while the adrenal gland was preserved. Seven days later, the right kidney was completely excised, resulting in a 5/6 Nx. Samples were collected 4 weeks after the procedure. In model 2, 2.5 g of adenine was dissolved in 100 mL of saline to prepare a 2.5% adenine suspension. The suspension was administered orally at a dose of 200 mg · kg^−1^ · d^−1^ (0.8 mL · 100  g^−1^ · d^−1^) based on the body weight of the rats. Samples were collected 4 weeks after the administration. In model 3, after abdominal disinfection, a longitudinal incision of 1 cm was made on the left side to expose the left kidney and the upper segment of the ureter. The upper segment of the ureter was ligated at both ends with 4-0 absorbable sutures and then cut. Then the abdomen was closed layer by layer. Samples were collected 2 weeks after surgery. All rats were anesthetized with pentobarbital, and blood and kidney samples were collected. This study was approved by the Ethics Committee of Anhui University of Chinese Medicine (Approval No.: AHUCM-rats-2022071). All procedures were performed in strict accordance with the ethical principles for the use and care of animals.

### 2.2. Biochemical Assay and histopathology

Blood samples were collected from the abdominal aorta of rats, left to rest, and then centrifuged at 3000 rpm for 15 min to separate the serum. The levels of serum creatinine (SCR) and blood urea nitrogen (BUN) were measured in each group using enzyme-linked immunosorbent assay according to the instructions of the corresponding kits. To assess the characteristics of the RF model, hematoxylin–eosin (HE) and Masson staining were performed. Kidney tissues were fixed in 4% paraformaldehyde for 24 h, followed by gradient alcohol dehydration, xylene treatment, and embedding in paraffin. Thin paraffin sections of 3-µm thickness were prepared and baked at 70 °C for 30 min. HE and Masson staining were performed according to standard protocols, and the sections were sealed with neutral gum after staining.

### 2.3. RNA sequencing

Sequencing was performed at OE Biotech (Shanghai, China) using the Illumina sequencing platform. Total RNA was extracted using the mirVana miRNA Isolation Kit (Ambion, Austin, TX, USA) following the manufacturer’s protocol. RNA integrity was evaluated using the Agilent 2100 Bioanalyzer (Agilent Technologies, Santa Clara, CA, USA). The samples with an RNA integrity number ≥7 were subjected to subsequent analysis. The libraries were constructed using TruSeq Stranded Total RNA with Ribo-Zero Gold according to the manufacturer’s instructions. The libraries were then sequenced on the Illumina sequencing platform (HiSeqTM 2500 or other platform), and 150/125-bp paired-end reads were generated. Three samples were randomly selected from each group for sequencing.

### 2.4. Real-time quantitative polymerase chain reaction validation

Total RNA was isolated from kidney tissues using the TRIzol reagent (ShareBio, Shanghai, China) according to the manufacturer’s instructions. cDNA was synthesized from 1 μg of total RNA using the All-in-One First-Strand Synthesis MasterMix with dsDNase kit. Real-time quantitative polymerase chain reaction (RT-qPCR) was performed using a fluorescence quantitative PCR system (ABI) with Taq SYBR Green qPCR Premix (Universal) with gene-specific primers. The quantitative primers used in the analysis were designed and synthesized by Sangon Biotech (Shanghai, China), and are shown in Table 1. Three of each type of RNA were randomly selected, with half upregulated and half downregulated.

**Table 1.** Primers used in RT-qPCR.

Gene	Forward primer (5′ → 3′)	Reverse primer (5′ → 3′)
ID1	GAAGTGGTGCTTGGTCTGTC	CTCACTTTGCGGTTCTGAGG
Ryr1	CCTGTGGAGACCCTCAATGT	TCGCCATAGGACCAGTTGTT
Apoh	CGCCACCACCAATTCCTAAG	GCAATTGGGTCCAGTTTCCA
Adamts2	GGGAATGGGAGCCTTGTACT	GGTGTTGTTGTGCAGAGGTT
Pkp1	CTCCCAGGACGAGAAGTACC	CTCCTGAACACCAGATTGCG
CD44	GACTCAGGAGCCCACAACAA	TTGCCTCTTGGGTGGTGTTT
TCONS-00008870	CTAAGGCACATCGTTGGT	TTACACCTTGATCCCGTT
TCONS-00003937	TGGGGTCCAGTATGGCTGTA	TGAGAGCCCTTGGTTCGTTC
XR-001838600.1	CTGCACTGTCTCCGGATTCT	CCATCCACTCCAGACCCTTT
TCONS-00022918	TTACCACTCTCCCACCCAAC	AGTAAGGGCACCAGTGGAAG
XR-001836016.1	GAGATACAGGAGGCCAGCAT	CTGTTGCCTGACGCTAAGTC
TCONS-00032144	ACTACCTCATGGACCACTGC	AATAGGCTAGCAGGGTTGGG
circ-3140	TCATAGGAACTGTGTCTGACCT	TCCTCCAGCTTCCTGAGAGA
circ-4199	TAGCTGACGAAGAGGACACAGA	TCATTGCTGCCCTGGTGTTG
circ-4266	GGCAAGACATTGCATCAGAAGTT	AAGCGAAGGAACGGGCAA
circ-0798	AGCCGAGGATTAGCACTCAC	TCCTTTCTCCCTCCAAAGCG
circ-3359	GAAGCGCACCTCTCAGTCAG	CATTTTCCAAACTCGTGGTCTG
circ-1121	TATCCGCTCTGGCTGTGATG	TCTCCATTCAGACCTGCGTT

### 2.5. Construction of the ceRNA network

Differential expression analysis of mRNAs, lncRNAs, and circRNAs was performed using DESeq software. The filtering criteria for selecting significantly differentially expressed (DE) RNAs were set as *P* < 0.05 and |log_2_ fold change| ≥ 1. To control the false discovery rate (FDR) associated with multiple testing, Benjamini-Hochberg correction was applied [23]. All miRNA target predictions were conducted using miRanda v3.3a, with known rat miRNA sequences retrieved from miRBase. To enhance specificity and reliability, stringent parameters were applied, including a minimum alignment score of 150, a maximum free energy threshold of −30 kcal/mol, and strict seed region matching. The MuTaME [24] method was used to calculate the ceRNA score for the predicted regulatory relationships between miRNAs and mRNAs, between miRNAs and lncRNAs, and between miRNAs and circRNAs. Hypergeometric distribution was used to calculate the *P*-value [25]; a smaller *P*-value indicates that the shared miRNAs between two ceRNAs (e.g., mRNA and its target) have a significant regulatory relationship. The correlation between mRNA and lncRNA or circRNA was calculated using the Pearson correlation coefficient (pearsonr), with a filtering criterion of an absolute correlation coefficient (|*r*|) ≥ 0.80 and a *P* ≤ 0.05. Only positively correlated RNA pairs were retained for further analysis. The intersection of these results with the ceRNA score calculations was obtained. From the ceRNA analysis results, the top 100 mRNA-lncRNA pairs were selected, 200 miRNA-mRNA-lncRNA triplet relationships were extracted based on these pairs, and a network diagram was drawn. Network visualization was implemented using Cytoscape.

### 2.6. Dual-luciferase reporter assay

siRNA, reporter gene vectors, and luciferase plasmids were constructed and transfected into NRK-52E cells in accordance with the manufacturer’s instructions. The cells were lysed using passive lysis buffer at room temperature with shaking for 15 min. Firefly luciferase activity was measured using LAR II, and Renilla luciferase activity was measured using Stop&Glo. The results were normalized by calculating the ratio of firefly to Renilla luciferase activity (F/R).

### 2.7. RNA fluorescence in situ hybridization

Cy3-labeled TCONS_00008870 probe (CTTTGGAACCTCGTGGCATAA) and circRNA_3140 probe (TGGGTGATACTGGGCACCTATATTA) were obtained from GenePharma (Shanghai, China). The distribution of lncRNA TCONS_00008870 and circRNA_3140 in NRK52E cells was detected using a fluorescence in situ hybridization (FISH) kit (Beyotime, China). FISH was performed according to the manufacturer’s instructions. Briefly, the cells were first treated with a complex digestive solution containing proteinase K and pepsin, denatured at 75 °C for 5 min, then hybridized in a wet and dark environment at 42 °C overnight using the probe hybridization solution. Afterward, the cells were washed with 0.4 Saline-Sodium Citrate (SSC) and Phosphate-Buffered Saline (PBS), and the nuclei were counterstained with 4’,6-Diamidino-2-Phenylindole (DAPI). Finally, images were captured using a fluorescence microscopy.

### 2.8. Western blot experiment

Total protein was extracted, separated by electrophoresis, and transferred to a membrane. After blocking with skimmed milk for 2 h, the membrane was incubated overnight with an anti-Adamts2 primary antibody (Bioss, Woburn, MA, USA, Bs-5858R, BD11112385, 1:500), followed by an HRP-conjugated secondary antibody for 2 h. Protein signals were detected using enhanced chemiluminescence, and the relative expression of Adamts2 was calculated using β-actin as an internal control.

### 2.9. Immunofluorescence

Paraffin-embedded rat kidney tissue sections were subjected to deparaffinization and rehydration using a series of xylene and ethanol incubations. The sections were then fixed with 4% paraformaldehyde for 15 min, followed by permeabilization with 0.5% Triton X-100 at room temperature for 20 min. For antigen retrieval, the sections were heated in sodium citrate buffer (10 mM, pH 6.0) by microwaving at 98 °C for 10 min. Afterward, the sections were blocked with 10% normal goat serum for 1 h, followed by overnight incubation with Adamts2 antibody (Bioss, Bs-5858R, BD11112385, 1:500). After washing, the sections were incubated with Alexa Fluor 488 fluorescent secondary antibodies for signal detection, and the nuclei were counterstained with DAPI. Finally, the stained sections were visualized using a fluorescence microscopy.

### 2.10. Immunohistochemistry

We recruited 15 patients with CKD and 5 patients who underwent nephrectomy due to renal cell carcinoma from the First Affiliated Hospital of Anhui University of Chinese Medicine. The non-tumor, normal kidney tissue from the nephrectomy patients was collected as control. Renal biopsy specimens from CKD patients and the control kidney tissues from nephrectomy patients were collected for immunohistochemical studies. Paraffin sections were blocked with serum and incubated overnight at 4 °C with the primary antibody anti-Adamts2 (Bioss, Bs-5858R, BD11112385, 1:500). Following primary antibody incubation, the sections were incubated with a biotinylated secondary antibody (horseradish peroxidase, 1:100 dilution) at room temperature for 1 h, followed by staining with 3,3′-diaminobenzidine tetrahydrochloride. ImageJ software was used to quantitatively analyze the staining density of Adamts2 in immunohistochemical staining.

### 2.11. Enrichment analysis

To explore the potential functions of the DE ncRNAs, three samples from each group were selected for gene ontology (GO) enrichment analysis and Kyoto Encyclopedia of Genes and Genomes (KEGG) pathway enrichment analysis. GO analysis included the categories ‘Biological Process (BP)’, ‘Cellular Component (CC)’, and ‘Molecular Function (MF)’ (www.geneontology.org). Differentially expressed genes (DEGs) were investigated mainly using KEGG (www.genome.jp/kegg/).

### 2.12. RNA–RBP network

To examine overlapping lncRNAs (19) and circRNAs (247), interactions with RBPs were predicted to construct the RNA–RBP network using RBPmap (http://rbpmap.technion.ac.il/). The predicted interactions were ranked according to *P*-values in ascending order, and the top 500 unique interactions were selected to generate the network diagram. The final network was visualized using Cytoscape for clear representation.

### 2.13. Statistical analysis

Statistical analysis was performed using DEseq software, and GraphPad Prism 8 was used to generate bar graphs. All data are presented as mean ± standard error. The expression levels of each ncRNA and mRNA were represented as the logarithm (log2) of the fold change (FC) obtained from RT-qPCR analysis. Differential expression of mRNAs and ncRNAs was defined as *P* < 0.05 with |log2 FC| ≥ 1. *P* < 0.05 was considered statistically significant. All experiments were repeated at least three times.

## 3. Results

### 3.1. RF model evaluation

**Figure 2.**
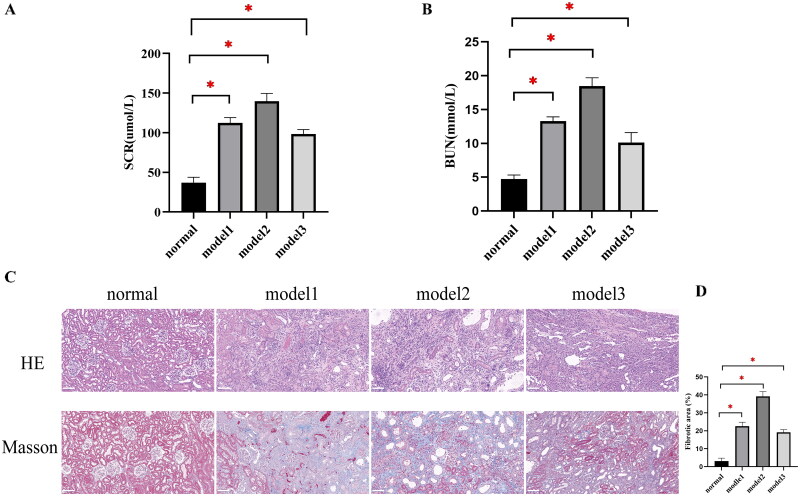
Validation of the RF models in SD rats. (A) Comparison of SCR levels in each group of rats. (B) Comparison of BUN levels in each group of rats. (C,D) HE and Masson staining of renal tissues in each group of rats. Scale bar = 100 μm. ^*^P < 0.0.05 compared with the normal group.

As outlined in the previous section, we established one normal group and three RF model groups of 10 rats each. Analysis of the groups revealed significant changes in renal function indicators in the three model groups compared with the normal group (*P* < 0.05), with elevated SCR and BUN levels in the model groups (Figure 2(A,B)). Histopathological analysis also revealed significant tissue changes. In the normal group, the glomeruli appeared full, the capillary loops were clearly structured, the renal tubules were arranged in an orderly fashion, and no apparent abnormalities appeared in the renal interstitium. In contrast, the 5/6 NX, adenine-induced, and UUO model groups showed glomerular and tubular atrophy, multifocal interstitial fibrosis, and tubular dilation (Figure 2(C,D)).

### 3.2. Whole-transcriptome sequencing and differential RNA identification

**Figure 3.**
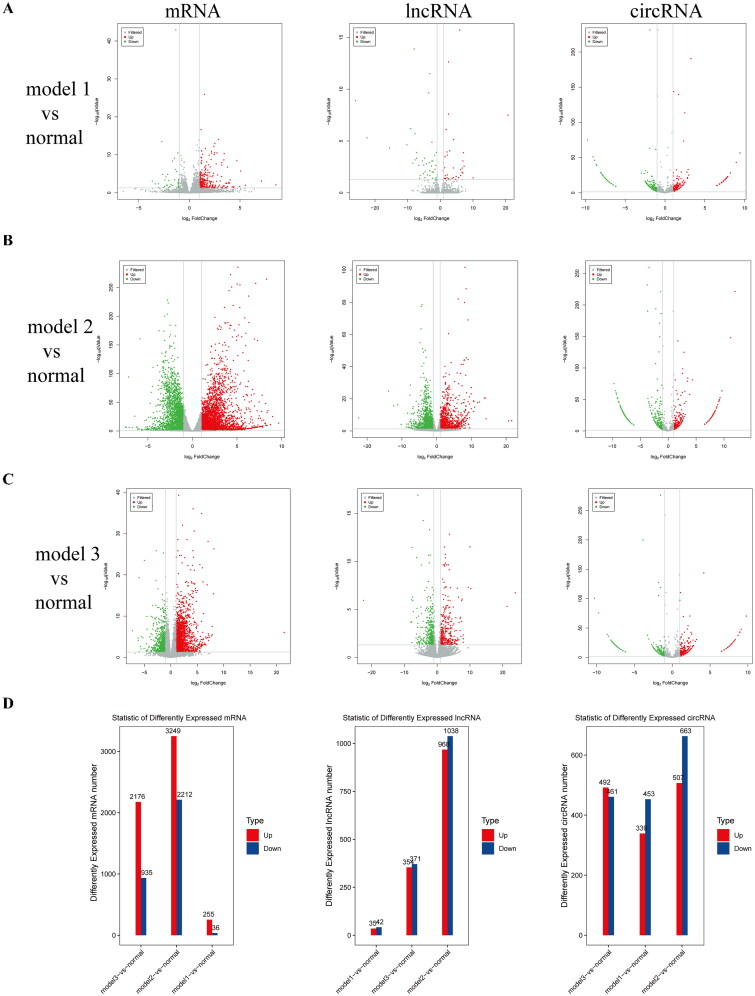
Expression profiles of mRNAs, lncRNAs, and circRNAs. (A–C) Volcano plots of significantly DE mRNAs, lncRNAs, and circRNAs between each model (model 1, model 2, and model 3) and the normal group. (D) Bar charts illustrating the upregulated and downregulated mRNAs, lncRNAs, and circRNAs in the three models compared with the normal group.

**Figure 4.**
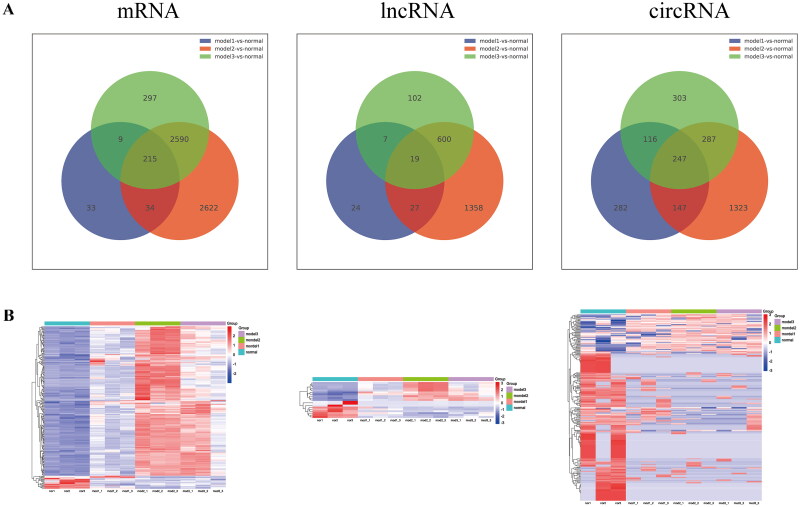
Venn diagram (A) and heatmap (B) of overlapping mRNAs, lncRNAs, and circRNAs among the three groups.

After whole-transcriptome sequencing, the expression levels of lncRNA, circRNA, and mRNA were estimated using the fragments per kilobase of exon per million fragments mapped method (Supplementary Table 1). Based on the filtering criteria of *P* < 0.05 and |log2 FC| ≥ 1, 291 differential mRNAs (255 upregulated, 36 downregulated), 77 differential lncRNAs (35 upregulated, 42 downregulated), and 792 differential circRNAs (339 upregulated, 453 downregulated) were identified in model 1 (5/6 Nx). In model 2 (adenine), 5461 differential mRNAs (3249 upregulated, 2212 downregulated), 2006 differential lncRNAs (968 upregulated, 1038 downregulated), and 1170 differential circRNAs (507 upregulated, 663 downregulated) were identified. In model 3 (UUO), 3111 differential mRNAs (2176 upregulated, 935 downregulated), 725 differential lncRNAs (354 upregulated, 371 downregulated), and 953 differential circRNAs (492 upregulated, 461 downregulated) were identified (Figure 3(A–D)). Analysis of the intersection of the differential genes from the three models revealed 215 overlapping differential mRNAs, 19 overlapping differential lncRNAs, and 247 overlapping differential circRNAs. These data are presented in the Venn diagram and heatmap in Figure 4.

### 3.3. RT-qPCR verification

**Figure 5.**
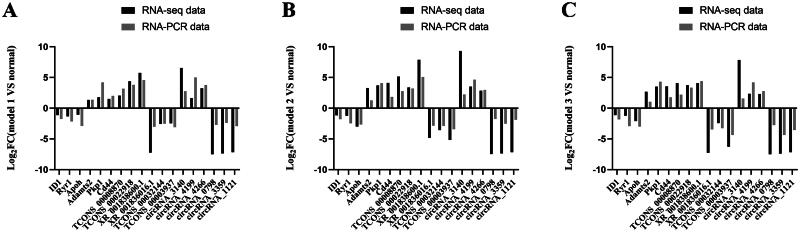
Validation of DE mRNAs, lncRNAs, and circRNAs in the three models using RT-qPCR. (A) Model 1 vs normal; (B) Model 2 vs normal; and (C) Model 3 vs normal.

To assess the reliability of the RNA-seq data, we selected six mRNAs, six lncRNAs, and six circRNAs from the overlapping DE RNAs across the three models, half of which were upregulated and the other half downregulated in each category, and validated them in all three models. The RT-qPCR results showed that the expression trends of the DE RNAs were consistent with the RNA sequencing results, indicating that the sequencing data were reliable (Figure 5(A–C)).

### 3.4. Construction of the ceRNA regulatory network

We next identified the ceRNAs, including the mRNAs, lncRNAs, and circRNAs, involved in competing for MREs and constructed ceRNA networks associated with lncRNAs or circRNAs to reveal the regulatory interactions and potential biological functions among lncRNAs, circRNAs, miRNAs, and mRNAs.

#### 3.4.1. ceRNA network analysis of model 1

**Figure 6.**
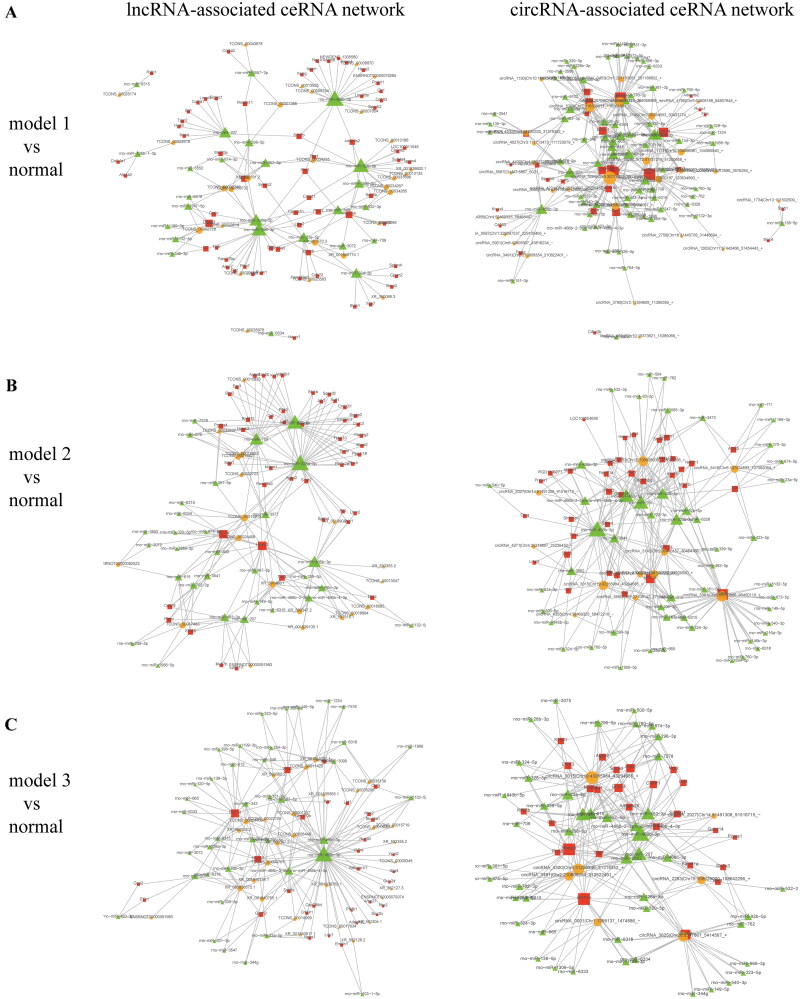
lncRNA-associated and circRNA-associated ceRNA networks in RF rats. (A) lncRNA-associated and circRNA-associated ceRNA networks in model 1 compared with the normal group. (B) lncRNA-associated and circRNA-associated ceRNA networks in model 2 compared with the normal group. (C) lncRNA-associated and circRNA-associated ceRNA networks in model 3 compared with the normal group.

In model 1, we identified 303 positively correlated lncRNA-miRNA-mRNA relationships and 614 positively correlated circRNA-miRNA-mRNA relationships (Supplementary Table S2). On the basis of these relationships, we constructed the lncRNA-associated ceRNA network and the circRNA-associated ceRNA network (Figure 6(A)). In the lncRNA-associated ceRNA network, lncRNA (ENSRNOT00000079265) forms a significant competitive regulatory relationship with the target genes Plakophilin 1 (Pkp1) and Adam metallopeptidase with thrombospondin type 1 motif 2 (Adamts2) by sequestering the shared miRNA (rno-miR-466b-3p) through its MRE. In the circRNA-associated ceRNA network, the top 10 results based on ceRNA score indicated that circRNA (circRNA_4356) has a significant positive competitive regulatory relationship with potassium calcium-activated channel subfamily N member 4 (Kcnn4) through the shared miRNA (rno-miR-466b-3p) MRE.

#### 3.4.2. ceRNA network analysis of model 2

In model 2, we identified 202,810 positively correlated lncRNA-miRNA-mRNA relationships and 614 positively correlated circRNA-miRNA-mRNA relationships (Supplementary Table S3). Based on these relationships, we constructed the lncRNA-associated ceRNA network and the circRNA-related ceRNA network (Figure 6(B)). In the lncRNA-associated ceRNA network, rno-miR-466b-3p and rno-miR-423-5p are the core regulatory nodes. In the circRNA-associated ceRNA network, rno-miR-483-3p and rno-miR-466b-3p serve as key regulatory nodes, connecting multiple circRNAs and mRNAs.

#### 3.4.3. ceRNA network analysis of model 3

In model 3, we identified 36,406 positively correlated lncRNA-miRNA-mRNA relationships and 13,338 positively correlated circRNA-miRNA-mRNA relationships (Supplementary Table S4). Based on these relationships, we constructed the lncRNA-associated ceRNA network and the circRNA-associated ceRNA network (Figure 6(C)). In the lncRNA-associated ceRNA network, multiple lncRNAs form competitive regulatory relationships with human immunodeficiency virus type I enhancer binding protein 3 (Hivep3) through the shared MRE of rno-miR-466b-3p, with rno-miR-466b-3p being the most important regulatory node. In the circRNA-associated ceRNA network, circRNA_3015 forms competitive regulatory relationships with Hivep3 *via* the shared MRE of multiple miRNAs, including rno-miR-466b-3p and rno-miR-483-3p.

#### 3.4.4. Core ceRNA network analysis

**Figure 7.**
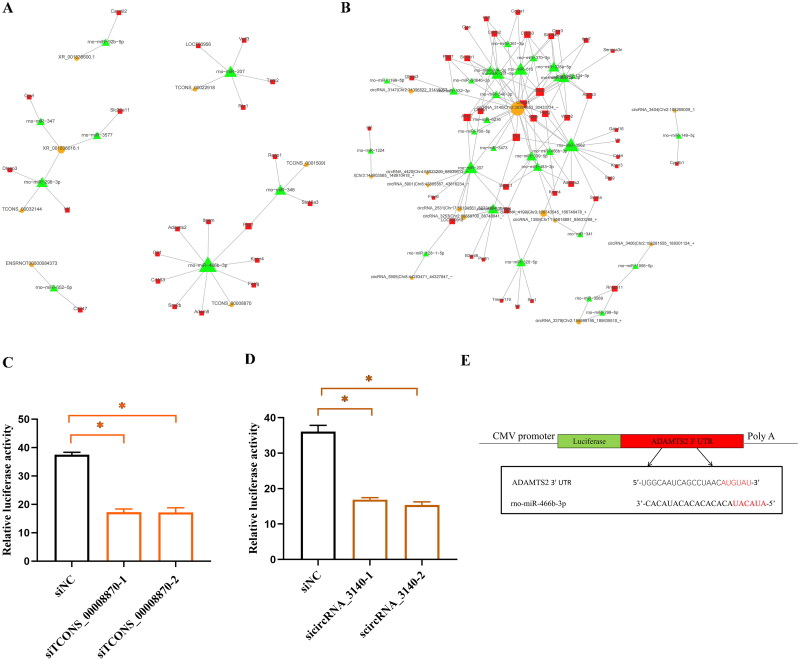
Core ceRNA networks constructed based on overlapping DE RNAs. (A) lncRNA-associated ceRNA network. (B) circRNA-associated ceRNA network. (C,D) Results of double luciferase experiment. NRK-52E cells were transfected with siTCONS_00008870 or sicircRNA_3140, Renilla luciferase plasmid, and firefly luciferase reporter plasmids harboring the Adamts2 3′ UTR. The ratio of firefly (F) to Renilla (R) in relative luciferase activity was plotted. siNC, siRNA with scrambled sequences; siTCONS_00008870, siRNA against lncRNA(TCONS_00008870); sicircRNA_3140, siRNA against the junction sites of circRNA(circRNA_3140). (E) Schematic diagram of miRNA (rno-miR-466b-3p) interaction with Adamts2 3′ UTR based on a luciferase reporter system. ^*^P < 0.05.

To identify conserved regulatory mechanisms shared among the three RF models, we performed an integrative analysis based on the ceRNA networks constructed for each model. Specifically, we identified 215 overlapping DE mRNAs, 19 overlapping DE lncRNAs, and 247 overlapping DE circRNAs across the three models. We selected 23 positively correlated lncRNA-miRNA-mRNA relationships and 49 positively correlated circRNA-miRNA-mRNA relationships (Supplementary Table S5) and constructed ceRNA networks based on these relationships. The lncRNA-associated ceRNA network contains 7 lncRNAs, 8 miRNAs, and 21 mRNAs, while the circRNA-associated ceRNA network includes 13 circRNAs, 29 miRNAs, and 41 mRNAs (Figure 7(A,B)). rno-miR-466b-3p is an important regulatory node in both ceRNA networks, where lncRNA (TCONS_00008870) and circRNA (circRNA_3140) form positive competitive regulatory relationships with Adamts2, Kcnn4, and other mRNAs through the shared MRE of rno-miR-466b-3p.

### 3.5. Dual-luciferase reporter assay validation

To confirm the core ceRNA regulation, we performed a dual-luciferase reporter assay in NRK-52E cells, selecting the top-scoring two lncRNA/circRNA-miRNA-mRNA axes from the lncRNA/circRNA-associated ceRNA networks for validation. The results indicated that knocking down lncRNA (lncRNA TCONS_00008870) and circRNA (circRNA_3140) reduced their competitive binding with miRNA (miR-466b-3p), thereby enhancing the binding between miRNA and mRNA (Adamts2), which ultimately led to downregulation of the target gene Adamts2 expression (Figure 7(C–E)). Thus, circRNA and lncRNA may function through the ceRNA network, but further experimental validation is needed to confirm the ceRNA regulatory mechanism.

### 3.6. Verification of ncRNAs and protein expression

**Figure 8.**
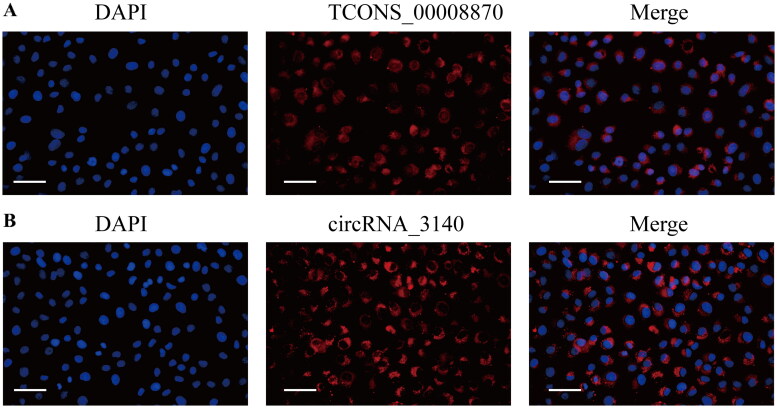
RNA-FISH results in NRK-52E cells. (A) Representative FISH image of TCONS_00008870 in NRK-52E cells. (B) Representative FISH image of circRNA_3140 in NRK-52E cells. Scale bar, 50 μm.

**Figure 9.**
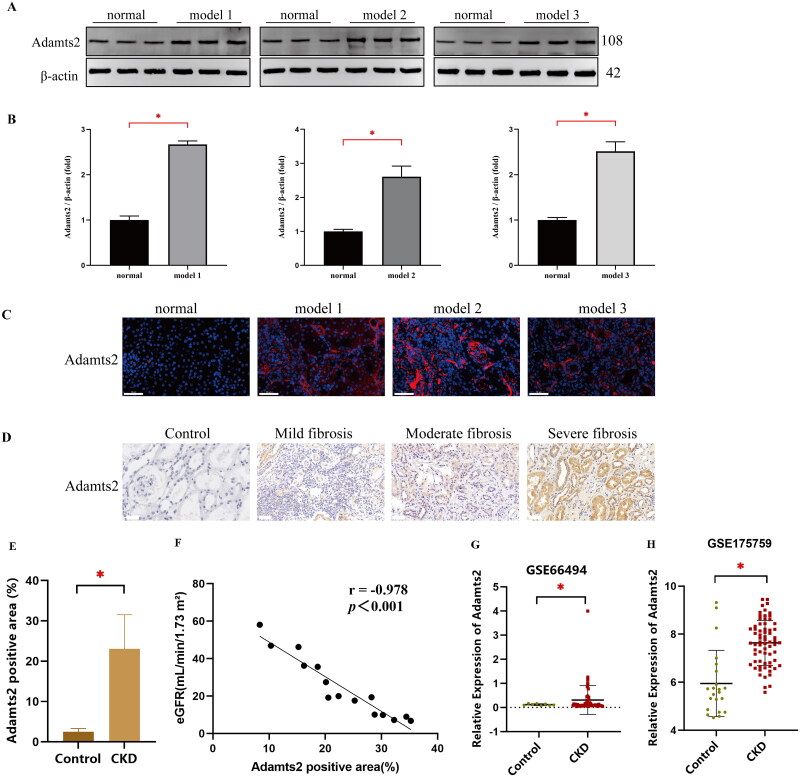
(A,B) Western blot analysis showed that Adamts2 protein expression was significantly upregulated in all three models (model 1, model 2, and model 3) compared with the normal group. (C) Representative immunofluorescence images of Adamts2 in rat kidney tissues from normal and three models. (D) Representative images of Adamts2 immunohistochemistry in control and CKD patients. (E) Quantitative results of Adamts2 immunohistochemistry in control and CKD patients. (F) Correlation analysis between Adamts2-positive area and eGFR in CKD patients (*n* = 15). (G) GSE66494: Microarray analysis showing Adamts2 expression in renal biopsy samples from CKD patients versus non-CKD controls. (H) GSE175759: RNA-seq analysis showing Adamts2 expression in tubulointerstitial tissues from patients with kidney diseases versus nephrectomy controls. Scale bar, 50 μm. All data are shown as mean ± SD, ^*^P < 0.05.

Previous validation established that the expression levels of lncRNA (lncRNA TCONS_00008870), circRNA (circRNA_3140), and mRNA (Adamts2) were elevated in all three models, consistent with the sequencing data (Figure 5). We further performed FISH for TCONS_00008870 and circRNA_3140 in NRK-52E cells. The results showed that both TCONS_00008870 and circRNA_3140 were predominantly localized in the cytoplasm of NRK-52E cells (Figure 8). Further analysis using western blot of rat kidney tissues showed that Adamts2 protein expression significantly increased in all three model groups but not in the normal group (*P* < 0.05) (Figure 9(A,B)). Additionally, we performed immunofluorescence staining for Adamts2 protein on kidney tissues from normal rats and all three model groups. The results demonstrated significantly increased expression of Adamts2 protein in all three model groups (Figure 9(C)). We also collected human renal biopsy specimens for immunohistochemistry of Adamts2 protein. Similarly, Adamts2 protein expression was significantly elevated in the kidney tissues of patients with CKD compared to the non-tumor, normal kidney tissue from the nephrectomy patients, which was used as a control (*P* < 0.05) (Figure 9(D,E)). Moreover, we collected clinical data from CKD patients and performed correlation analysis between Adamts2-positive staining area and estimated glomerular filtration rate (eGFR). The results indicated a significant negative correlation (Figure 9(F)), suggesting that Adamts2 upregulation is associated with renal function decline. To strengthen the external validation of Adamts2 expression, we further analyzed two publicly available transcriptomic datasets. The GSE66494 dataset includes microarray data from renal biopsy specimens of CKD patients and non-CKD controls. The GSE175759 dataset contains RNA-seq profiles from microdissected tubulointerstitial tissue of patients with various kidney diseases and nephrectomy controls. In both datasets, Adamts2 expression was significantly elevated in diseased samples compared to control (*P* < 0.05), consistent with our experimental findings (Figure 9(G,H)). These results from independent clinical cohorts further support the association of Adamts2 with RF and reinforce the reliability of the identified ceRNA regulatory axis.

### 3.7. Enrichment analysis

**Figure 10.**
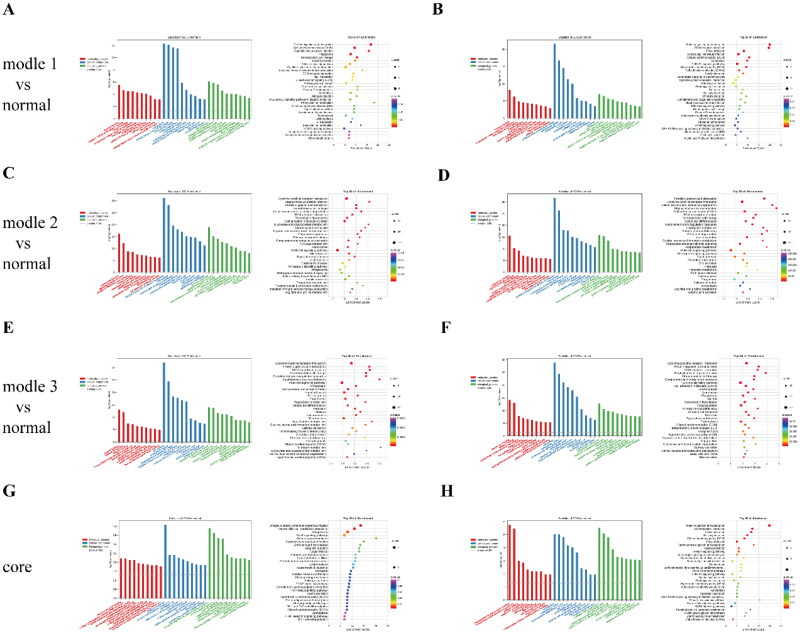
Bioinformatic analysis of the ceRNA networks in different models. (A) GO and KEGG pathway analysis of the lncRNA-associated ceRNA network in model 1 compared with the normal group. (B) GO and KEGG pathway analysis of the circRNA-associated ceRNA network in model 1 compared with the normal group. (C) GO and KEGG pathway analysis of the lncRNA-associated ceRNA network in model 2 compared with the normal group. (D) GO and KEGG pathway analysis of the circRNA-associated ceRNA network in model 2 compared with the normal group. (E) GO and KEGG pathway analysis of the lncRNA-associated ceRNA network in model 3 compared with the normal group. (F) GO and KEGG pathway analysis of the circRNA-associated ceRNA network in model 3 compared with the normal group. (G) GO and KEGG pathway analysis of the lncRNA-associated core ceRNA network. (H) GO and KEGG pathway analysis of the circRNA-associated core ceRNA network.

To reveal the functions of mRNAs involved in the lncRNA or circRNA-associated ceRNA networks, we performed GO and KEGG pathway enrichment analyses. The lncRNA- associated ceRNA network in model 1 is mainly involved in key phenomena and processes such as the ECM, cell adhesion, and ECM–receptor interactions (Figure 10(A)), while the circRNA-associated ceRNA network functions through the regulation of the ECM, cell adhesion, and the PI3K-Akt signaling pathway (Figure 10(B)). The lncRNA-associated ceRNA network in model 2 further includes key processes such as immune response and the tricarboxylic acid cycle (Figure 10(C)), while the circRNA-associated ceRNA network also involves functions such as fatty acid degradation and tryptophan metabolism (Figure 10(D)). The lncRNA-associated ceRNA network in model 3 is enriched in immune response and receptor-mediated endocytosis in addition to the aforementioned processes (Figure 10(E)), while the circRNA-associated ceRNA network is also enriched in ECM–receptor interactions and the PI3K-Akt signaling pathway (Figure 10(F)).

Based on the functions of mRNAs in the core ceRNA networks, we also performed GO and KEGG pathway enrichment analysis. We found that the Rap1 signaling pathway was enriched in the lncRNA-associated ceRNA network, while the ECM–receptor interaction and PI3K-Akt signaling pathways were enriched in the circRNA-associated ceRNA network (Figure 10(G,H)).

### 3.8. RNA–RBP interaction networks

**Figure 11.**
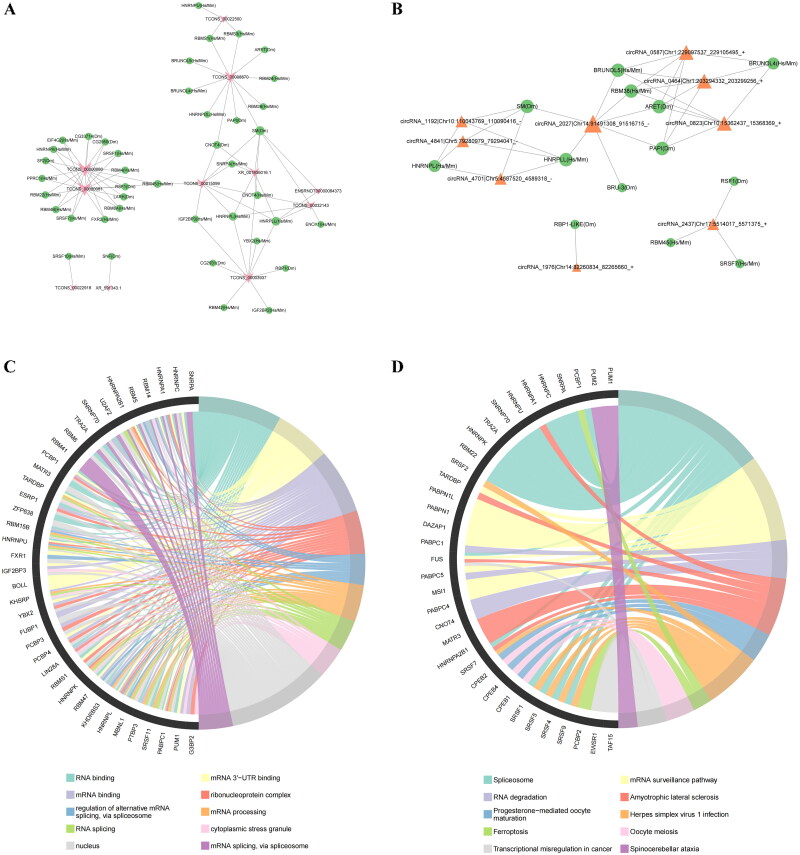
RNA–RBP network. (A) RNA–RBP network of the DEGs in the subnetwork of the lncRNA-associated ceRNA network. (B) RNA–RBP network of the DEGs in the subnetwork of the circRNA-associated ceRNA network. (C) GO enrichment of RBPs. (D) KEGG enrichment of RBPs.

RNA–protein interactions represent another functional mechanism of RNA. As investigating these networks aids in understanding the role of RBPs in disease onset and progression, we constructed RNA–RBP networks (Figure 11(A,B)). The lncRNA–RBP network included 11 lncRNAs and 41 predicted RBPs, and the circRNA–RBP network included 9 circRNAs and 13 predicted RBPs. When we performed KEGG and GO enrichment analysis to study these RBPs in more depth, we observed that RNA binding, RNA splicing, and the ferroptosis pathways were significantly enriched (Figure 11(C,D)).

## 4. Discussion

RF is a common pathological process in the progression of CKD [26], a global public health issue [27], and its inhibition may play a significant role in delaying the onset of end-stage renal disease. NcRNAs have been identified as key novel regulators of RF and are rapidly emerging as promising therapeutic targets with significant potential for diagnostic and prognostic evaluation [28]. As long as they contain the same MREs in their 3′ UTR, lncRNAs and circRNAs can function as sponges to adsorb miRNAs, thus reducing or enhancing the stability of target RNAs and regulating various physiological and pathological processes within the organism [29]. While prior studies have explored ncRNA-mediated regulation in individual models, a comprehensive cross-model approach remains lacking. Our study addresses this gap by integrating whole-transcriptome data from three distinct RF models to uncover shared ncRNA regulatory patterns. Notably, we identified a conserved ceRNA axis—TCONS_00008870/circRNA_3140–miR-466b-3p–Adamts2—suggesting a common fibrotic pathway regardless of initiating cause.

As it plays crucial roles in renal diseases by regulating gene expression, miRNA is emerging as a potential biomarker and therapeutic target [30]. In this study, we identified the core regulatory node miR-466b-3p, which previous research has shown targets histone deacetylase 7 (HDAC7) and negatively regulates its expression, affecting epigenetic modifications [31]. However, the potential role of miR-466b-3p in RF remains unexplored, and further studies are needed to validate its underlying mechanisms. Additionally, two other DE miRNAs have been extensively studied in relation to renal diseases. miR-423-5p promotes cell proliferation [32] and regulates glucose and amino acid metabolism in cancer cells [33]. Multiple studies have demonstrated that miR-483-3p plays a significant role in antifibrosis [34,35] and is aberrantly expressed in both *in vitro* and *in vivo* diabetic nephropathy models, where it induces apoptosis in renal tubular epithelial cells [36].

Targeted mRNAs of these miRNAs have been studied to reveal their specific roles in renal diseases. Adamts2, an ECM metalloproteinase, promotes myofibroblast activation and ECM accumulation by regulating the TGF-β signaling pathway and collagen processing, thereby exacerbating fibrosis [37–39]. Kcnn4, a calcium-dependent potassium channel, mediates cell proliferation in various cell types, including fibroblasts, and promotes RF [40,41]. Hivep3, a transcription factor, plays a role in regulating gene expression, particularly influencing cell differentiation, immune responses, and inflammation [42].

Interestingly, ECM-related processes and cell adhesion were repeatedly enriched across all three models, reinforcing the notion that these pathways represent core fibrotic mechanisms shared across distinct etiologies. Excessive ECM deposition is a hallmark of fibrosis [43], while altered cell adhesion may exacerbate the fibrotic process by influencing renal tubular epithelial–mesenchymal transition and fibroblast activation [44]. The significant enrichment of the ECM–receptor interaction pathway suggests that the ceRNA network associated with lncRNAs and circRNAs may play an important role in the initiation and progression of fibrosis by regulating the dynamic balance of the ECM. RAP1 can be activated by exchange protein directly activated by cyclic adenosine monophosphate to trigger the cAMP-dependent protein kinase A pathway, thereby affecting cell adhesion [45]. Additionally, Rap1 promotes macrophage activation, which exacerbates renal tubular epithelial cell death and contributes to RF [46]. In this study, the Rap1 signaling pathway, a crucial mechanism for regulating cell adhesion and ECM remodeling, was significantly enriched in the ceRNA network, indicating that it may further promote fibroblast activation, migration, and the progression of fibrosis through its regulation of integrin–ECM interactions.

The PI3K-Akt signaling pathway plays a critical role in regulating cell survival, proliferation, and metabolism [47]. Its multiple enrichments suggest that lncRNAs and circRNAs may regulate fibrosis-related cellular behaviors, such as fibroblast proliferation and anti-apoptosis, *via* this pathway. The cross-regulation of Rap1 and PI3K-Akt signaling may further participate in cell proliferation and anti-apoptosis, thus becoming an important mechanism in the regulation of fibrosis. This study is the first to reveal that the Rap1 signaling pathway may be involved in the molecular regulation of fibrosis through the ceRNA network, thereby expanding the understanding of the role of Rap1 in RF.

Integrated analysis of the three models showed that model 1 (5/6 Nx) focused more strongly on ECM-related functions, making it suitable for studying fibrosis and matrix remodeling in CKD. The immune response enrichment in models 2 and 3 demonstrated the important role of inflammation in fibrosis [48]. Specifically, the enrichment of metabolic pathways in model 2 suggests that the mechanism of gouty nephropathy is related to metabolic disorders, particularly abnormalities in fatty acid degradation and tryptophan metabolism, that may trigger immune responses and exacerbate renal injury. Moreover, the repeated enrichment of the ECM–receptor interaction, PI3K-Akt, and Rap1 signaling pathways across all models suggests that these pathways could serve as common regulatory mechanisms for fibrosis. This further highlights the role of the ceRNA network in these pathways, suggesting that ncRNAs may serve not only as diagnostic biomarkers for fibrosis but also potentially as therapeutic targets.

When we constructed lncRNA–RBP and circRNA–RBP networks, we observed that the RNA binding, RNA splicing, and ferroptosis pathways were significantly enriched in both networks. RBPs are involved in the regulation of various cellular processes, including RNA stability, transport, translation, and splicing [49]. During RF, abnormal RNA splicing or translation may lead to the overexpression or dysregulation of fibrosis-related genes, thereby accelerating the pathological process. Furthermore, activation of the ferroptosis pathway could be an important mechanism in RF [50]. The RNA–RBP network may exacerbate or inhibit ferroptosis by regulating the expression of genes related to iron metabolism. Therefore, RNA–RBP interactions not only regulate RNA functions but may also have a profound impact on the occurrence and development of RF by mediating specific cellular pathways.

Despite the valuable findings of this study, certain limitations should be acknowledged. While we identified and functionally validated a conserved ceRNA regulatory axis—TCONS_00008870/circRNA_3140–miR-466b-3p–Adamts2, the depth of functional validation remains limited. In particular, gene silencing experiments and *in vivo* phenotypic assessments have not yet been conducted. These will be essential components of future research to advance from correlative observations to causal mechanistic insights. Nevertheless, the consistent identification of this conserved regulatory axis across three distinct RF models provides a solid foundation for further mechanistic studies and the development of potential therapeutic interventions.

## 5. Conclusion

In summary, we conducted whole-transcriptome sequencing across three distinct models and identified a conserved ceRNA axis—TCONS_00008870/circRNA_3140–miR-466b-3p–Adamts2. This axis was functionally validated and is associated with key fibrosis-related pathways. Our findings highlight a shared regulatory mechanism in RF and provide a potential target for future therapeutic intervention.

## Supplementary Material

Supplementary Table S2.xlsx

Supplementary Table S1.xlsx

Supplementary Table S5.xlsx

Supplementary Table S3.xlsx

Supplementary Table S4.xlsx

## Data Availability

The authors can confirm that all relevant data are included in the article, which are open and transparent.
